# The Glocal Forest

**DOI:** 10.1371/journal.pone.0126117

**Published:** 2015-05-08

**Authors:** Efrat Seri, Elad Shtilerman, Nadav M. Shnerb

**Affiliations:** 1 Department of Physics, Bar-Ilan University Ramat-Gan, Israel; 2 Porter School of Environmental Studies, Tel Aviv University, Ramat Aviv, Israel; Ben-Gurion University of the Negev, ISRAEL

## Abstract

Spatial ecological patterns reflect the underlying processes that shape the structure of species and communities. Mechanisms like intra- and inter-specific competition, dispersal and host-pathogen interactions can act over a wide range of scales. Yet, the inference of such processes from patterns is a challenging task. Here we call attention to a quite unexpected phenomenon in the extensively studied tropical forest at the Barro-Colorado Island (BCI): the spatial deployment of (almost) all tree species is statistically equivalent, once distances are normalized by *ℓ*_0_, the typical distance between neighboring conspecific trees. Correlation function, cluster statistics and nearest-neighbor distance distribution become species-independent after this rescaling. Global observables (species frequencies) and local spatial structure appear to be interrelated. This "glocality" suggests a radical interpretation of recent experiments that show a correlation between species' abundance and the negative feedback among conspecifics. For the forest to be glocal, the negative feedback must govern spatial patterns over *all* scales.

## Introduction

An understanding of the forces that govern the dynamics of populations and communities is one of the major challenges of contemporary ecology [1]. Besides its practical importance for management and conservation, the apparent excess biodiversity in systems like tropical forests, coral reef and freshwater plankton poses a thought-provoking conceptual problem, as it seems to violate the competitive exclusion principle [2]. The numerous attempts to resolve this puzzle [3–9], and the hot debates these studies generated, reveal that a clear understanding of these mechanisms is still lacking.

One of the most useful probes for such systems, and in particular for communities of sessile species, is the set of statistical properties related to spatial patterns characterizing the focal community. This feature may reflect dispersal limitations [10, 11], competition [12–15], interactions with pathogens and predators [16], environmental filtering [17] and many other circumstances [18]. Accordingly, numerous analyses of aggregation, patchiness and structure have appeared during the last decade, most of which have been utilizing the data from the spatially explicit, long-term monitoring of a few tropical forests provided by the Center for Tropical Forest Science (CTFS). Virtually all these works implemented various techniques of point pattern analysis [19], taking every tree as a point in space and retrieving quantities like the correlation function from the matrix of distances between individuals.

Here we report the results of a few point pattern analyses for >1cm trees and understory plant in the Barro-Colorado plot [20–22]. The only difference between our work and previous studies is that, for any given species, we have normalized the distances between trees by the species-specific fundamental length-scale *ℓ*_0_, defined via $\ell_{0}=\sqrt{A/N_{i}}$, where A is the plot area and *N*_*i*_ is the number of conspecific trees (or understory shrubs) in that area (i.e., the abundance of the i-th species). Notably, after this rescaling it appears that all species have a statistically identical spatial structure (at least up to the length scale of the plot), with minor differences that appear mainly below *ℓ*_0_. This outcome imposes a very strong constraint on the models that may explain or describe spatial distributions. Our results suggest that a reasonable model has to be glocal, i.e., to allow the overall abundance of a species (a global property) to dictate the local structure or vice versa. This feature arises in many simple models of forest dynamics, such as the Poisson and the fractal models considered in S1 File, but these models fail to account for the empirical patterns observed in realistic systems.

A possible mechanistic interpretation of our results may be related to recent experiments of Mangan et. al. [23]. These authors have analyzed the negative feedback between conspecific trees for 6 species in the BCI forest, pointing out that the strength of this feedback varies among species and is a good predictor of the relative abundance of a species. Similar results were obtained in [24–26].

Our results suggest a far-reaching generalization of these findings, namely, that the negative feedback is (for *almost all* species) the dominant factor in governing the statistical properties of spatial patterns. Other effects, and in particular interspecific interactions [15] and dispersal limitations [27], appear to be relatively weak.

## Methods

Along this paper we are implementing three methods of statistical assessment aimed to characterize two dimensional spatial patterns. The first measure, the distribution of distances between a tree and its nearest neighbor (NNDD), provides information on the short-distance scales; it is related, for example, to the typical distance between a parent and offspring, or in general to the dispersal kernel. The second spatial statistic is the spatial correlation function, which provides further information regarding the intermediate length scales (10–50 meters). As these two methods do not allow for separation of length-scales, we present a third measure which is based on multiscale cluster statistics. All these measures are well-documented in the literature [28–31]; the novelty of our study has to do with the implementation of these statistics with distances rescaled by the species-specific typical length-scale *ℓ*_0_.

Our analysis is focused on the spatial structure of trees in a tropical forest, the data used, as stated above, was taken from the first general census of the Barro-Collorado Island 50-ha plot (other censuses were also analyzed, yielding similar results). In S1 File we compared the results obtained from the BCI forest to the results of the same statistical analysis when applied to "simulated forests", i.e., computer-generated point patterns that reflect a few popular mechanistic models used to explain several aspects of tropical forest spatial patterns.

## Results

**Fig 1:**
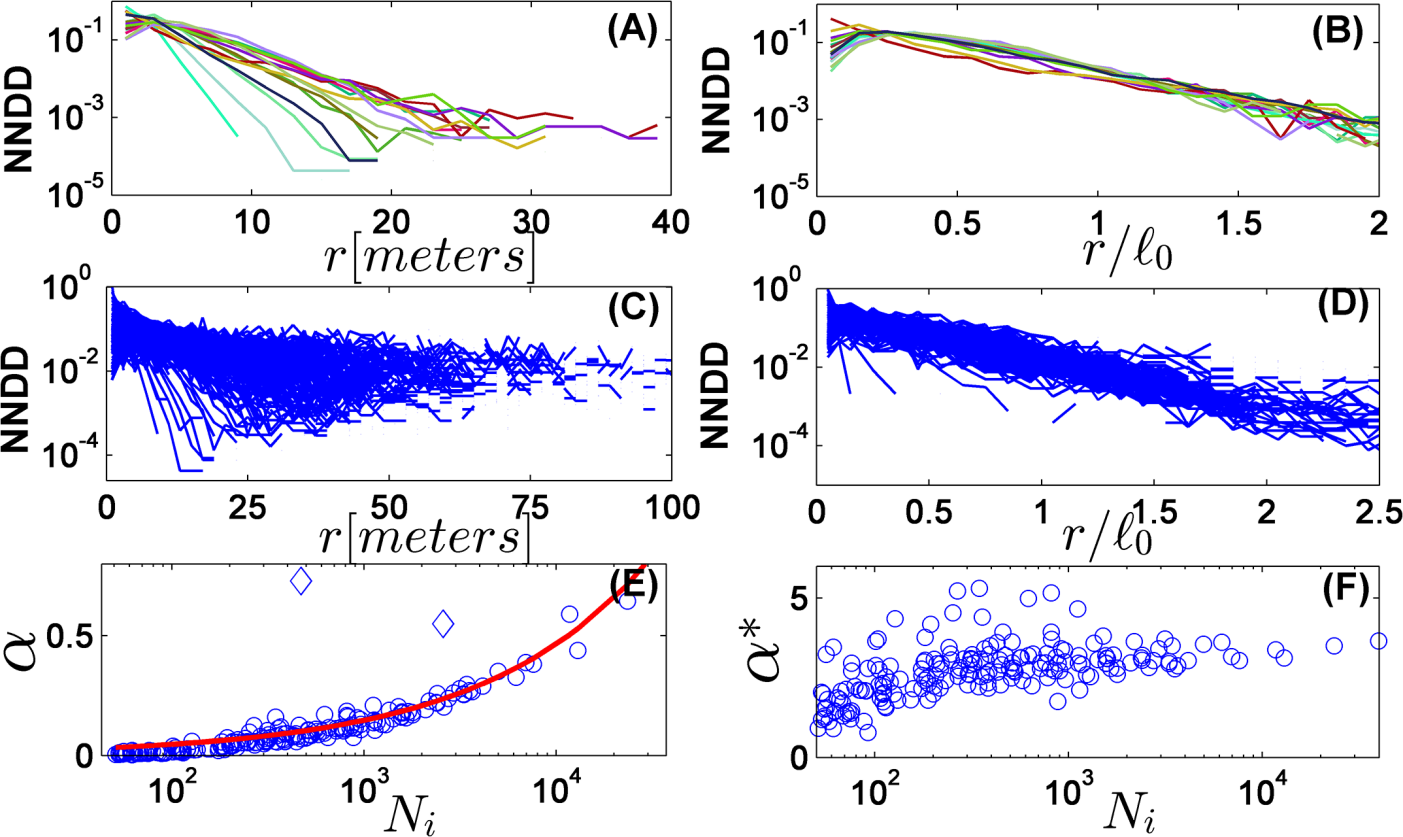
Nearest neighbor distance distribution (NNDD) in the BCI tropical forest. For every species, the graph presented is a normalized histogram of the distances from every tree to its closest conspecific tree in the forest [28]. In panel (A) the data is presented for the 15 most abundant species on a semi-log scale. The decay at long distances is clearly exponential, but the slope varies strongly among species. Panel (B) shows the same data when distances are rescaled by *ℓ*_0_, showing a good data collapse in the exponential regime. Panels (C) and (D) are the same graphs for all species with >50 individuals in the forest. The data is noisier, but the pronounced features are the same. In panel (E), the slope *α* in the exponential regime is plotted against the abundance of the species *N*_*i*_, showing a $\sqrt{N_{i}}$ dependence as expected (red line, notice the logarithmic scale). This correlation is much weaker, and almost disappears for *N*_*i*_ < 150 when the rescaled data is analyzed (panel (F), α* is the slope measured in rescaled coordinates).

Let us start with one of the standard measures of spatial structure, the nearest-neighbor distance distribution [28]. In Fig 1 (left panels) we plot this quantity for the 15 most abundant species, and for all species with more than 50 individuals in the forest. The statistics is quite good, and one observes, beyond the scale of a few meters, an exponential decay of the distribution over four orders of magnitude. *P*(*r*), the chance to find the nearest neighbor tree at a distance *r*, follows (at *r* > 0.5*ℓ*_0_) *P*(*r*) ∼ exp(−*α r*) where, as expected, *α* is an increasing function of the abundance. Such an exponential decay in two spatial dimensions is very interesting by itself and its origin is not clear; in any case, the results clearly exclude a Gaussian decay of the NNDD (as predicted for a Poisson forest) and a crossover from Gaussian to power-law one finds for negative-binomial spatial distributions.

Here we would like to highlight another property: once the same graphs are plotted for the rescaled distance *r* / *ℓ*_0_ (Fig 1, right panels), the exponent α* ≡ *α ℓ*_0_ becomes (almost) abundance independent for *N*_*i*_ > 150 species. The clear correlation between the exponent and the abundance practically disappears after rescaling, and the data collapse is quite impressive.

For *N*_*i*_ < 150, Fig 1(f) indicates an average increase of α* with abundance. This may be an artifact of the non-universal behavior at short length scales, and in any case it is difficult to assess the statistical significance of the results when the number of points is so small. However, if this effect is significant it suggests that, with respect to this measure, rare species are less aggregated than the frequent species, contrary to the conclusion of the very influential paper by Condit et. al. [29] and in agreement with [32].

To emphasize the novelty of this collapse, we show in S1 File, section 2, the corresponding figures for four mechanistic models that were used in the literature to account for the spatial deployment of forests: A Poisson process, spatial neutral dynamics with mixed local-global recruitment kernel (MLGK), which is similar to the Cox process, spatial neutral dynamics with a Cauchy (fat-tailed) kernel and the fractal structure (random Cantor set) suggested in [33].

As shown in S1 File, the Poisson forest indeed shows a data collapse in the rescaled coordinates (since it admits only one length scale which is determined by the abundance), but the function *P*(*r*) is a Gaussian, not exponential, as expected for random point patterns [28]. The two processes with a finite kernel (MLGK and Cauchy) admit two scales: one is determined by the kernel, the other by the abundance. As a result, they both fail to yield a collapse and the plots of *α* and α* vs. abundance show a clear trend. A fractal structure admits, again, a single length scale, but now this scale has nothing to do with the abundance (the basic scale may reflect, say, spatial heterogeneity), so the collapse occurs in the "wrong" plot, when the length scale is not normalized.

**Fig 2:**
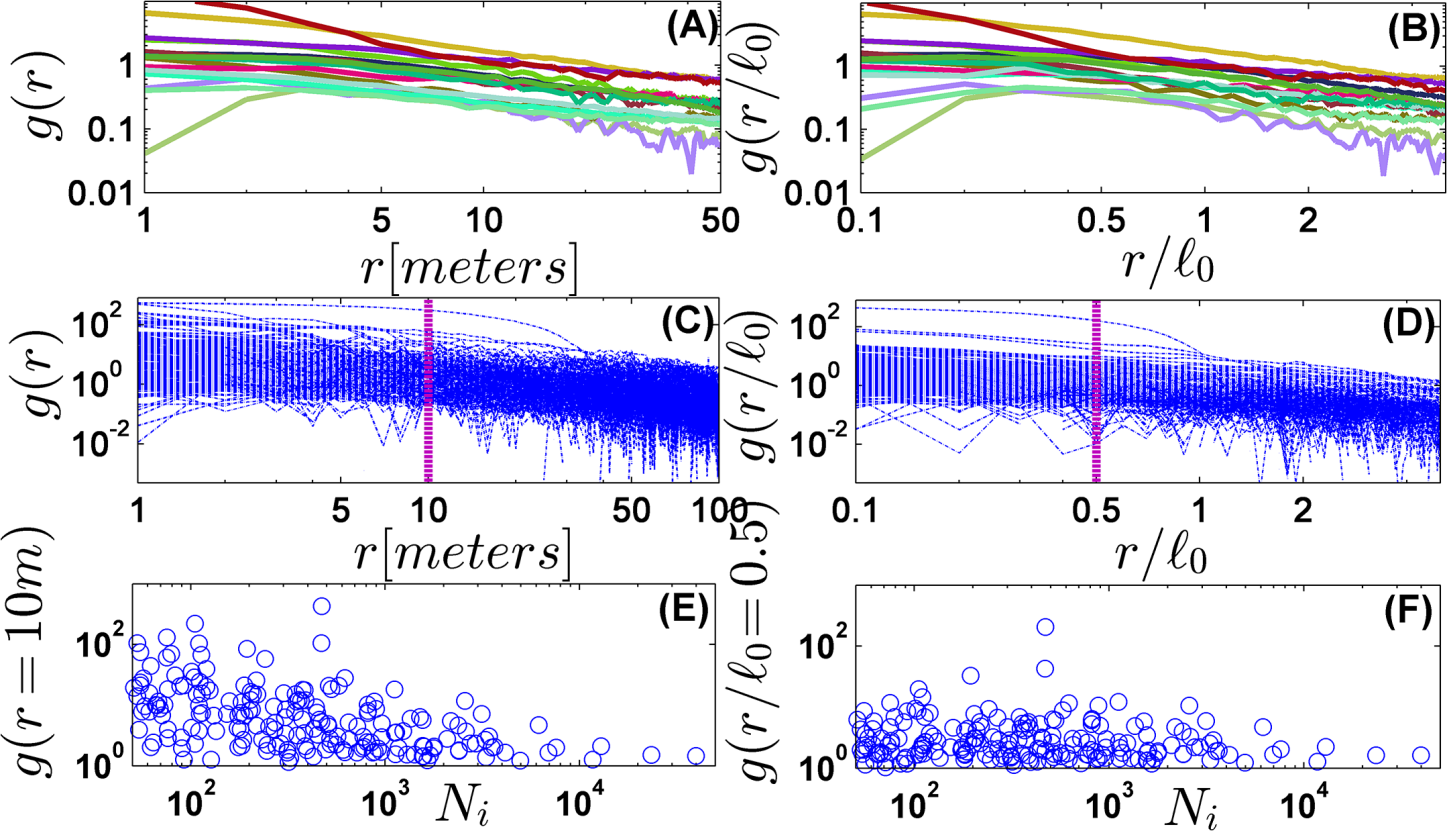
The correlation function. For each individual *i*, two quantities are measured: *s*_*i*_(*r*), the number of conspecific trees in a ring of inner distance (from the focal tree) *r* and outer distance *r* + Δ*r*, and *A*_*i*_(*r*), the fraction of this ring area that is included in the 50-ha plot. The correlation function $g(r)\equiv \frac{\sum s_{i}(r)}{N_{i}\sum A_{i}(r)}-1$ (the sum runs over all focal-specie's trees) measures the information about density fluctuations embodied in the observation of a tree at *r* = 0. *g*(*r*) = Ω(*r*) – 1, where Ω is the relative neighborhood density defined in [29], and is zero for a homogenous Poisson process. At long distances the decay of the correlations is described quite faithfully by a power law [33] (panel A, 15 most abundant species, panel C, all species). The short-distance correlation tends to be higher when a species is rare. This property is demonstrated in panel (E), where the height of the correlation function at *r* = 10*m* is plotted against the abundance, in parallel with Fig 2 of [29]. The rescaled plots (panels B and D) have the same slopes, since a power law is scale independent, but the crossover to the power law behavior is close to *r*/*ℓ*_0_ = 1 for almost all species. Moreover, the association between abundance and correlation becomes non-significant in the rescaled plots, as seen in panel (F), showing the height of *g*(*r* / *ℓ*_0_ = 0.5) vs. *N*_*i*_. Pearson correlation coefficient in panel (E) is 0.46, with p-value less than 0.01, while for (F) the coefficient is 0.12 and the p-value is 0.1.

A similar effect of rescaling is revealed when the correlation function *g*(*r*) is considered. This function (also known as Ω-ring statistic, or the radial derivative of Ripley's K-function) was implemented in [29] to show that rare species in the tropical forest are more aggregated than common species. The results of [29] are illustrated again in Fig 2(E), showing a general trend towards lower correlations for more frequent species. After rescaling (Fig 2, right panels) this relation becomes non-significant. Since the decay of correlations above *r* > 0.5 *ℓ*_0_ resemble a power-law (in agreement with [10, 11, 33]), the rescaling of length does not affect the slope. Still, the absolute height of the correlation function is independent of the abundance when the comparison is made at the same value of *r* / *ℓ*_0_.

The corresponding graphs in S1 File, section 3 show that the mechanistic model cannot yield these features. In a Poisson forest the correlations are independent of the distance (up to noise and finite size effects), in the two dispersal models the low-abundance species appear to be more clustered, and the same property characterizes also the fractal forest.

**Fig 3:**
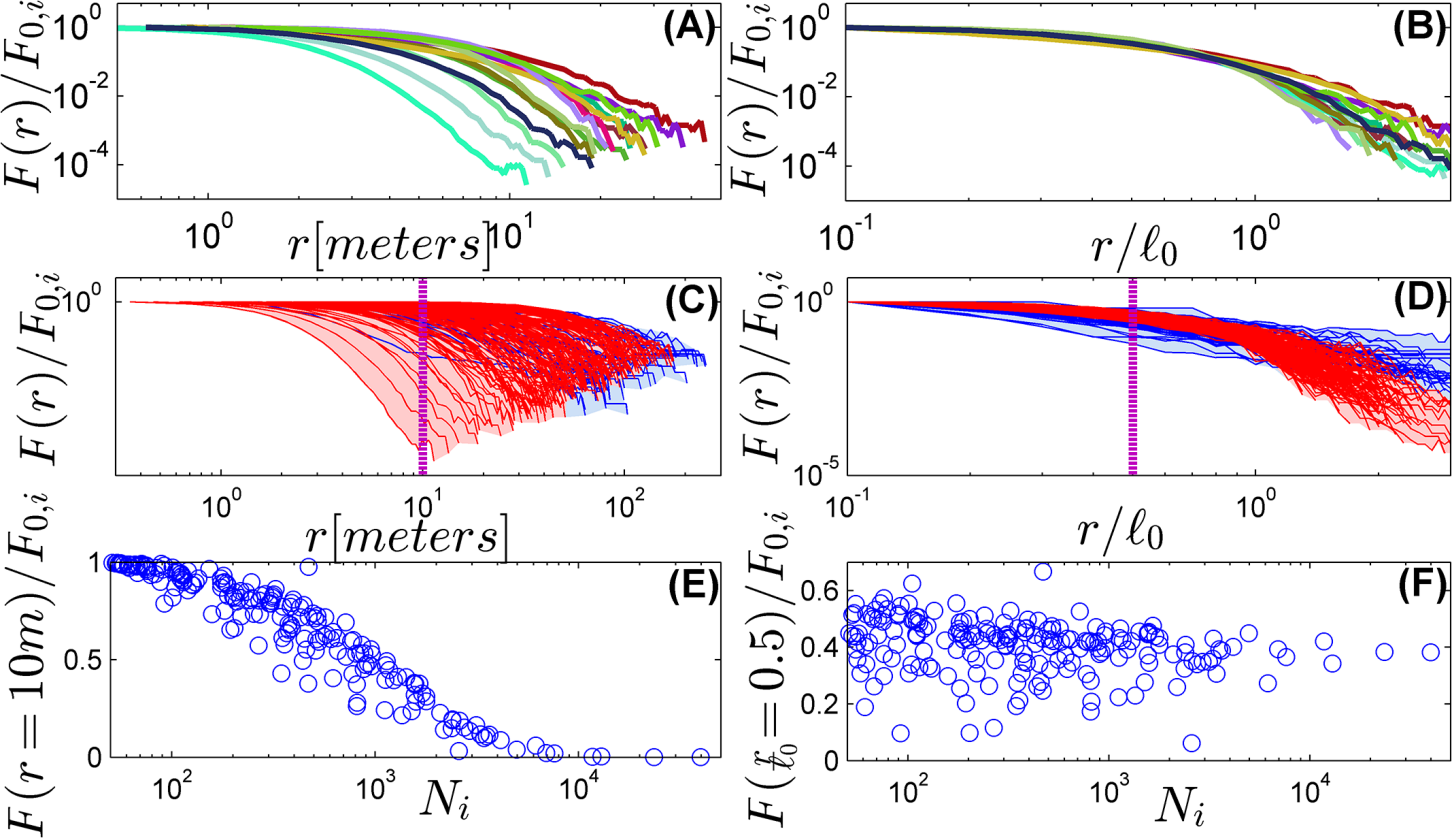
Separating spatial scales using patch statistics. The two dimensional 50-ha plot map was covered by a grid of *ℓ* × *ℓ* squares, every square is "black" if it contains at least one tree of the focal species, otherwise it is "white". *F*(*ℓ*), the number of clusters at each resolution level, is normalized by *F*_0,*i*_, the number of clusters for the minimal value of *ℓ* used, *F*_0,*i*_ → *N*_*i*_ as *ℓ* → 0. Panels (A) and (C) show *F*(*ℓ*)/*F*_0,*i*_ vs. *ℓ* for the 15 most abundant species (A) and for all species with more than 50 individuals in the forest (C). Panel (E) depicts *F*(*ℓ* = 10*m*)/*F*_0,*i*_ vs. *N*_*i*_ (a cut along the purple dashed line in panel (C)), emphasizing the abundance dependence of the result. Panels (B) and (D) show the same analysis when *F*(*ℓ*)/*F*_0,*i*_ is plotted vs. *ℓ*/*ℓ*_*0*_. The data collapses (we colored red 158 out of 193 species (82%), to emphasize that most of the width is due to a small number of exceptional species, see S1 File, section 5 and the correlation with abundance disappears (panel F, showing *F*(*ℓ* = 0.5*ℓ*_*0*_)/*F*_0,*i*_ vs. *N*_*i*_).

Both NNDD and the correlation function have a limited ability to separate length scales: these are probability distribution functions, and the normalization condition dictates anti-correlations between their values at short and long distances. As pointed out recently by [34], one would like to decompose variance scale by scale. To address this requirement we provide a third piece of evidence in Fig 3. Here the aggregation is characterized by the total number of tree clusters, with a varying grid scale *ℓ*. The two-dimensional map of the plot is covered by a mesh of *ℓ* × ℓ squares, each square colored black if it contains at least one tree of the focal species. Two black squares are in the same cluster if they are connected by a path of nearest neighbor black sites (see a detailed description in [30, 31]). The total number of different clusters, *F*(*ℓ*), approaches *N*_*i*_ when *ℓ* → 0 and converges to 1 for large *ℓ*. Counting the number of clusters at different spatial resolutions Fig 3 demonstrates, again, a quite good data collapse and vanishingly small correlation between the number of clusters and the species abundance in the rescaled data.

The figures in section 4 of S1 File demonstrate again that none of the mechanistic models imitates the real forest. The Poisson forest shows, as expected, a data collapse, but it deviates strongly from the empirical results, as there is no real clustering in a Poisson forest, the decay of *F*(*ℓ*) is much slower. The fractal forest data collapses, if any, in the non-normalized diagram, and the two dispersal models fail to yield a collapse.

## Discussion

All the indicators we have analyzed here are pointing at a quite surprising feature: the spatial structure of all tree species (up to a few exceptions) is almost identical, when measured with respect to the species specific length scale *ℓ*_0_. Such a feature emerges trivially in a Poisson forest (if *N*_*i*_ trees from the i-th species are randomly distributed in the forest, there is only one length scale $\ell_{0}=\sqrt{A/N_{i}}$ and the spatial aggregation characteristics will become *N*_*i*_-independent after rescaling by *ℓ*_0_) or in a "lattice" forest, where trees of every species are located on the vertices of a two-dimensional squared lattice with a lattice constant *ℓ*_0_. However, these two models are irrelevant since the aggregation in the forest is well-known to be stronger than Poisson [29], let alone of a lattice. Indeed, all our parameters deviate substantially from the Poisson/lattice limits; still the forest admits only a single scale.

On the other hand, it seems that every non-Poissonian mechanistic model of forest dynamics must admit at least one typical, species specific length scale associated either with the recruitment kernel, i.e., with the distribution of distances between mother and offspring (encapsulating the dispersal kernel and the chance of a seedling to capture an open slot) or with spatial heterogeneity. Our results show that this "local" scale, whatever it is, dictates (or is dictated by) the "global" scale *ℓ*_0_ associated with the overall density of the focal species in the forest. This puts a severe restriction on the space of possible models and, in fact, the property of glocality is not a part of *any* of the models we are familiar with, from neutral dynamics [10, 11, 35] to tradeoffs [9] to niche models. In all these mechanistic theories the local dynamics has nothing, or almost nothing, to do with the overall abundance of a species.

Former results, like the excess positive correlations of rare species observed in [29], may indeed reflect the dominance of a single length scale. The correlation function *g*(*r*) quantifies the information one has about the density fluctuations at a distance *r*, given the presence of a tree at the origin. If the rescaled measure *g*(*r*/*ℓ*_0_) is roughly the same for all species, it implies that the information for frequent species (small *ℓ*_0_) falls faster on real scale, rendering the infrequent species more aggregated.

It was already pointed out in [29], that trees of some exceptional species form circular clumps, apparently reflecting the effect of dispersal limitation. The spatial patterns of these species, like *Rinorea sylvatica*, *Anaxagorea panamensis* and *Bactris major* (see spatial patterns in S1 File, section 5) must admit at least two length scales—one associated with the cluster size, the other with the inter-patch distance. These exceptional species are not glocal, and indeed appear as outliers in Figs 1–3. For example, the two irregular points (diamond markers) in Fig 1(E) correspond to *Rinorea sylvatica* and *Bactris major*, where the value of *α* for *Anaxagorea panamensis* is so high that it cannot be seen in the frame presented here. The same phenomenon is evident in Figs 2 and 3. Moreover, the correlation function for these species clearly reveals a crossover between two slopes, as opposed to the single power-law for "standard" species. Still, the number of exceptional species is relatively small.

We believe that this glocality is not a peculiarity of the BCI forest, but a characteristic of other forests as well. Unfortunately we were not successful in obtaining access to the spatially resolved data of the other homogenous forest in the CTFS system in Pasoh, Malaysia. Hopefully other groups will have the opportunity to compare the rescaled spatial structure in different locations. Anyhow, at least any model for the BCI data should allow a glocal interrelation between scales.

Apparently, glocality requires a species specific mechanism that carries information along scales. Such a mechanism may act either top-down (e.g., when the overall density controls the dispersal kernel since the typical movement of a species specific animal disperser is proportional to the distance to the nearest neighbor conspecific individual) or bottom-up, for example, by negative feedback which reduces the probability of conspecific seedlings to survive in the proximity of an adult tree [34].

Indeed, Mangan et. al. [23] have demonstrated experimentally this mechanism, attributing it to plant-soil feedback mediated by soil biota and further showed that the feedback is a good predictor of the abundance of different species in the plot. As pointed out in the introduction, glocality may emerge from this mechanism, provided that the dominant factor that controls spatial patterns is the strength of "repulsion" between conspecific trees. If every species has its own "exclusion radius" due to intraspecific negative feedback, but otherwise the interspecific competition is uniform (neutral), the emerging pattern has to be glocal. However, this is a strong requirement, as it implies that all other length scales, like those involved in dispersal limitation and inter-specific competition have only a minor effect on the spatial deployment of the forest.

If true, our findings and the work of [23] suggest a model in which every species has its own mutual exclusion radius. Such a model may also explain the global correlations in the abundance of taxonomic groups [36]. Still, the role of competition in shaping community assembly and spatial structure is not clear. One may imagine a model in which competitive superiority is balanced by stronger negative feedback [as in the recent work of [37]] or neutral dynamics, but at this point we do not know under what conditions these dynamics do not alter the statistical properties of a spatial patterns.

## Supporting Information

S1 File Supplementary material. (DOCX)
