# Supplementary material for: The Glocal Forest
Source: PLoS One. 2015 May 8;10(5):e0126117. doi: 10.1371/journal.pone.0126117 (PMC4425529; doi:10.1371/journal.pone.0126117)
Supplement: S1 File — (DOCX) [file pone.0126117.s001.docx]

Supplementary material

# The mechanical models and simulation procedures

The results presented in the main text were extracted from the spatial deployment of trees and undercanopy in the BCI forest (the results used in this paper are of the first census). Here we present results obtained using the same set of analyses, when applied to a few *simulated* forests, each represents a popular mechanistic model which is implemented in the literature in order to explain the spatial structure of forests and other systems. To check for a data collapse and its (in)dependence on abundance, we have simulated a single species dynamics for each of the models below, on a plot of size 500x1000m^2^ (which is the size of the BCI plot), until the process reaches the prescribed abundance. The results for species with 40000, 20000, 10000, 4000, 2000, 1000 and 500 individuals were analyzed and compared. For some technical complications we have used a different set of numbers for the fractal forest (see 4 below), but there is no reason to think that it may change any general features of the results.

The models considered in this supplementary are:

1. **Poisson forest**. This is the simplest model, assuming that there is no spatial correlation between the mother tree and its offspring. Although the recruitment kernel must depend on the distance, this model becomes accurate when the linear size of the surveyed plot is much smaller than the typical length associated with the recruitment.
2. **MLGK (Cox-like) forest**: The Cox process is a result of a two-stage random mechanism. To build a Cox forest of N trees one choses m points (centers) at random, and place N/m trees (again at random) within a distance r from every center. To make the process slightly more realistic, we have implemented here a neutral dynamics with mixed local-global kernel, a model that we have used in a recent paper analyzing the spatial structure of the BCI plot. ([Seri et al. 2012](#_ENREF_1)). Starting with a single individual from the focal species, the neutral dynamics is implemented. In every elementary timestep two individuals are picked at random and the offspring of the (randomly selected) first replaces the second, see ([Volkov et al. 2003](#_ENREF_2)). Once the first individual is picked, the second is chosen at random from its 2-meters neighborhood with probability 1-μ and from the whole plot with probability μ.

The process continues until the desired number of individuals (the species abundance) is obtained, all other details are given in ([Seri et al. 2012](#_ENREF_1)). The limit μ=1 corresponds to the Poisson forest, for smaller μ-s every population is made of a random (Poisson) collection of clusters of individuals. In the simulation here we have used μ=0.1, as this value yielded the best (although unsatisfactory) fit to the BCI data in ([Seri et al. 2012](#_ENREF_1)).

1. **Cauchy forest**: is generated using the same neutral dynamics algorithm [see again ([Seri et al. 2012](#_ENREF_1))], but now the recruitment kernel is Cauchy, i.e., the probability that the descendent of a tree feels a gap at a distance r is given by:

$$K(r)=\frac{1}{\pi\gamma}\frac{1}{1+r^{2}/\gamma^{2}}.$$

Here $\gamma$ is the characteristic spatial scale of the kernel. In this type of kernel, there is no specific distance that separates global dispersal from local; rather, the probability decreases slowly with r. In ([Seri et al. 2012](#_ENREF_1)) we showed that the clusters obtained from a neutral process with this kernel fit quite nicely the BCI forest data.

1. **A Fractal** forest: to simulate a forest with a fractal structure, we have implemented the random Cantor set algorithm suggested as a model for tropical forests by (Green (2000)). Starting with a 2x2 array, each cell is chosen to be empty with probability P or is chosen to be "active" with probability 1-P. Each of the active cells is then divided into 4 equal squares and the process is iterated. The active sites of the last iteration are the focal species trees.

We have stopped the process when the forest reaches the size of 1024x1024 cells, using P=0.75. Implementing a few realizations of the same algorithm, we were able (due to the randomness of the process) to generate a few sets of focal species trees, sets that have the same fractal structure but different abundance. For the analysis presented below we have used realization with *n*  "trees" where $n\approx$40000, 20000, 10000, 4000 and 2000.

In the following sections we show the results obtained when we applied our measures to the various simulated forests. The panels of every figure correspond to the two upper panels [(A) and (B)] and the two lower panels [(E) and (F)] of the figures shown in the main text.

# Nearest-neighbor distance distribution (NNDD)

The NNDD for species with different abundance will give a data collapse when the distances are normalized by l_0_ if this is the only length scale in the forest. This is clearly true for a Poisson process but Figure 2.1 indicates that P(r) is a *Gaussian* (this is a known feature of random point-patterns in two dimensions), not the exponential distribution that characterizes the empirical data.

For the MLGK model (Figure 2.2) one can observe the crossover to Poisson statistics at long distances, but the short distance data does not collapse, and the Cauchy simulation (Figure 2.3) do not have a collapse region at all. Finally, the NNDD for a fractal forest (Figure 2.4) does collapse, but this occurs in the non-normalized (left) graph, since all fractals have the same basic length scale.


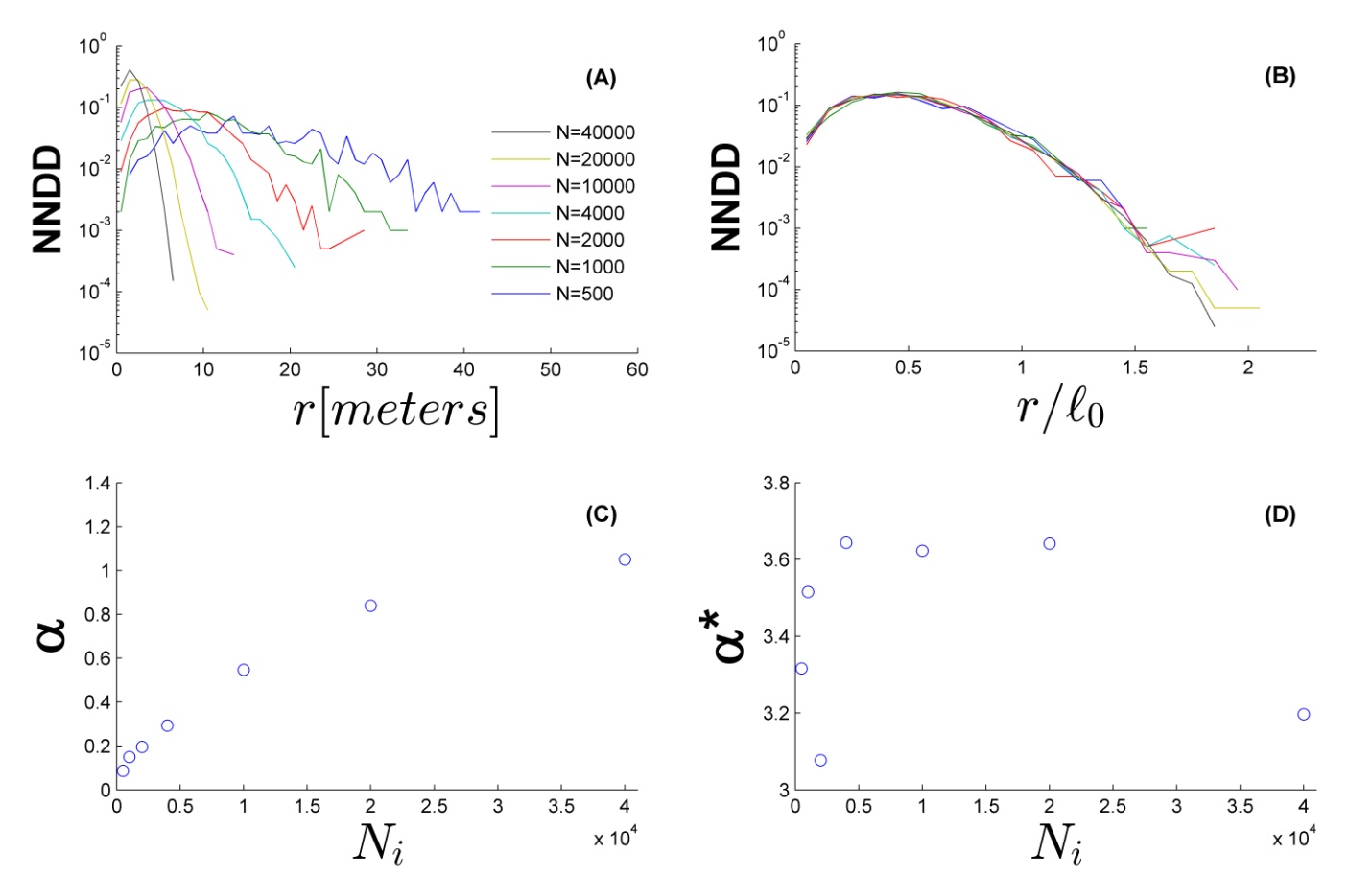


Figure 2.1: NNDD for Poisson distribution. (A) vs. the real distance r (to be compared with Fig. 1A of the main text). (B) vs. the normalized distances r/l_0_ (to be compared with Fig. 1B of the main text). (C) The slope of NNDD as a function of the species abundance N for (A) (to be compared with 1E), (D) :same as (C) but for the normalized curves in (B) (in parallel with panel 1F in the main text). In panels (C) and (D) the "slope" of the tail was measured as if the decay is exponential, just to show the results that correspond to the analysis presented in the main text. Indeed, of course, the decay is Gaussian so there is no reasonable way to compare the two systems.


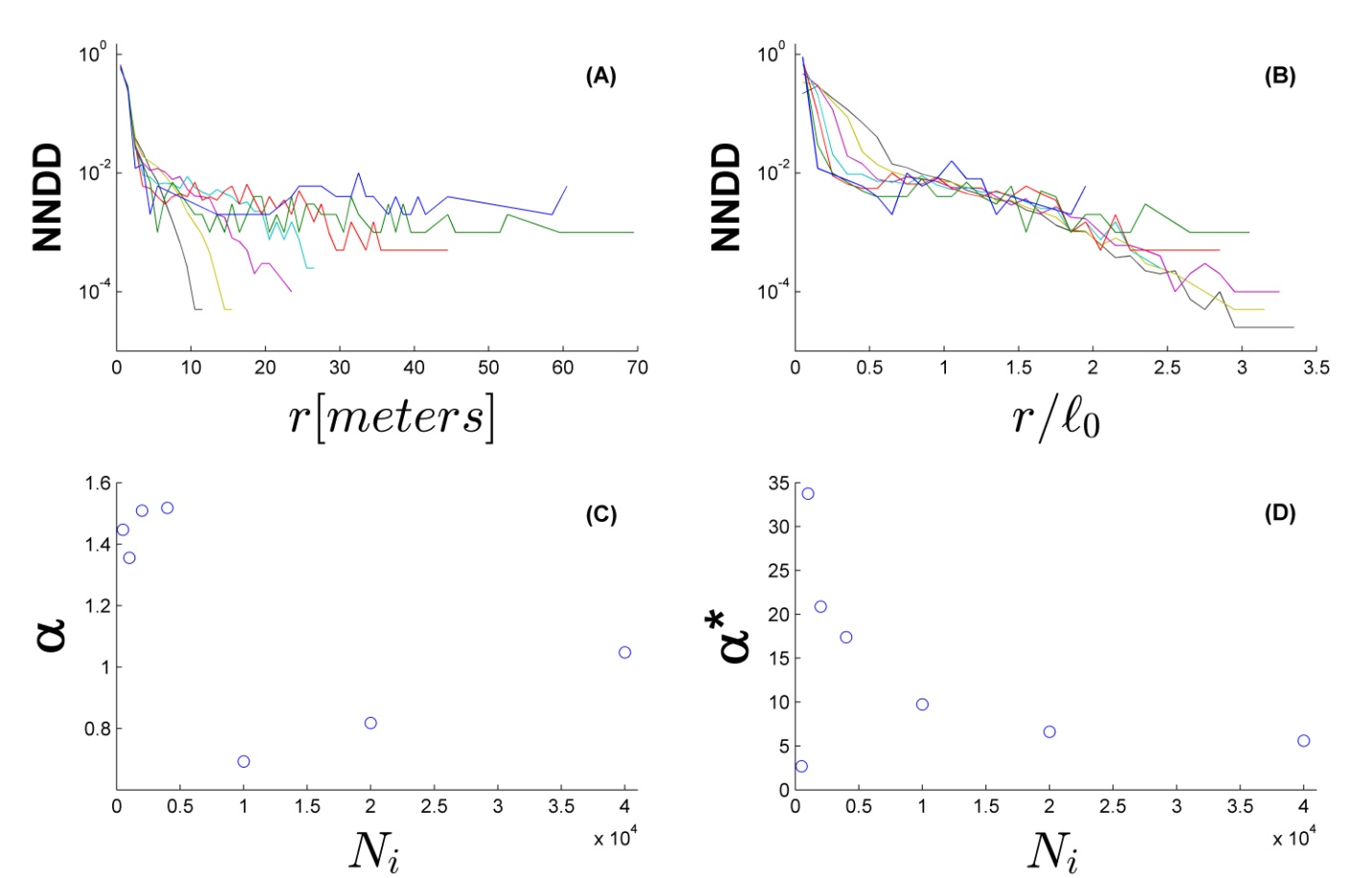


Figure 2.2: Same as fig. 2.1 but for MLGK (µ=0.1).


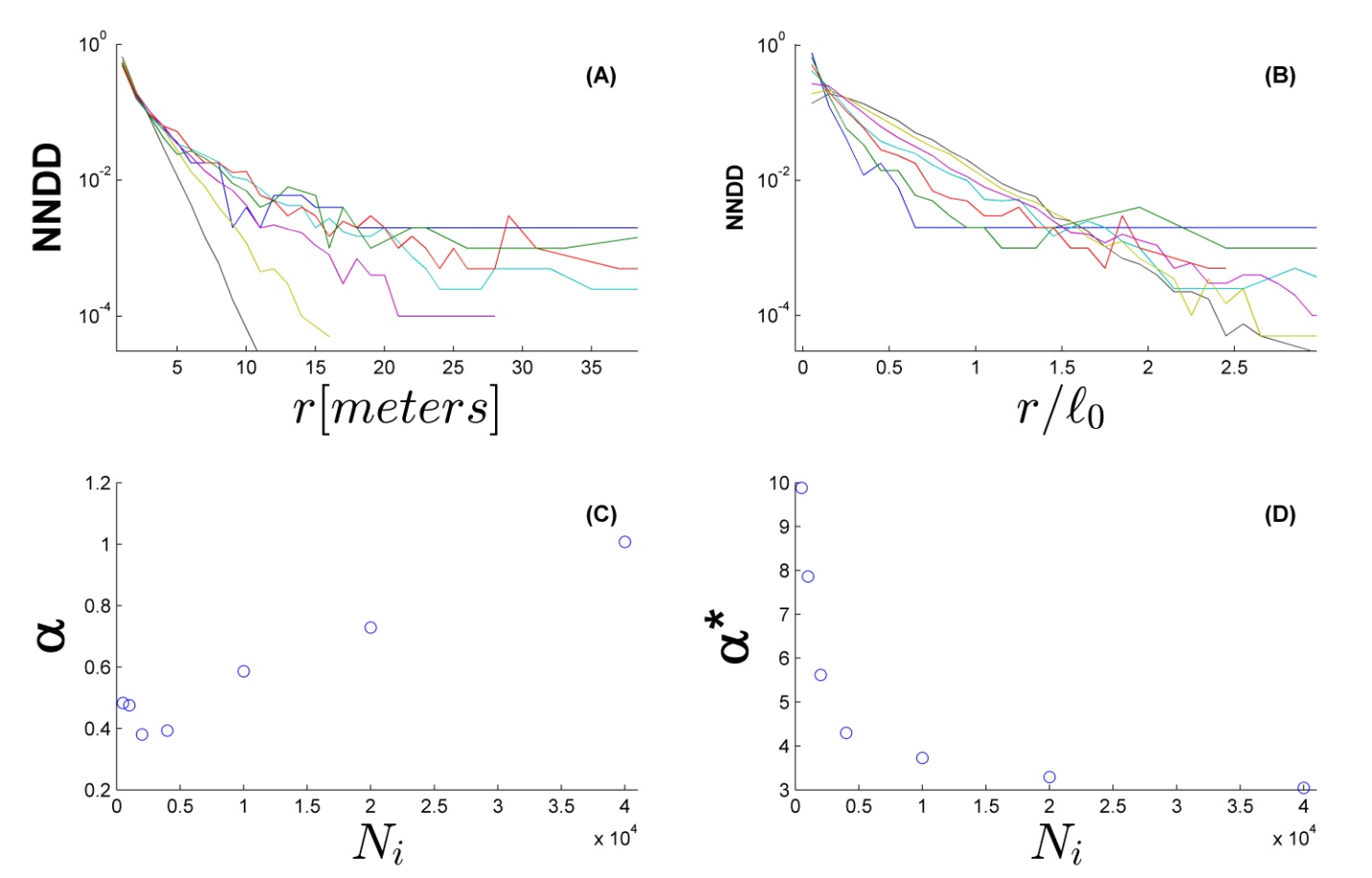


Figure 2.3: Same as fig. 2.1 here for the Cauchy kernel (ɣ=20)


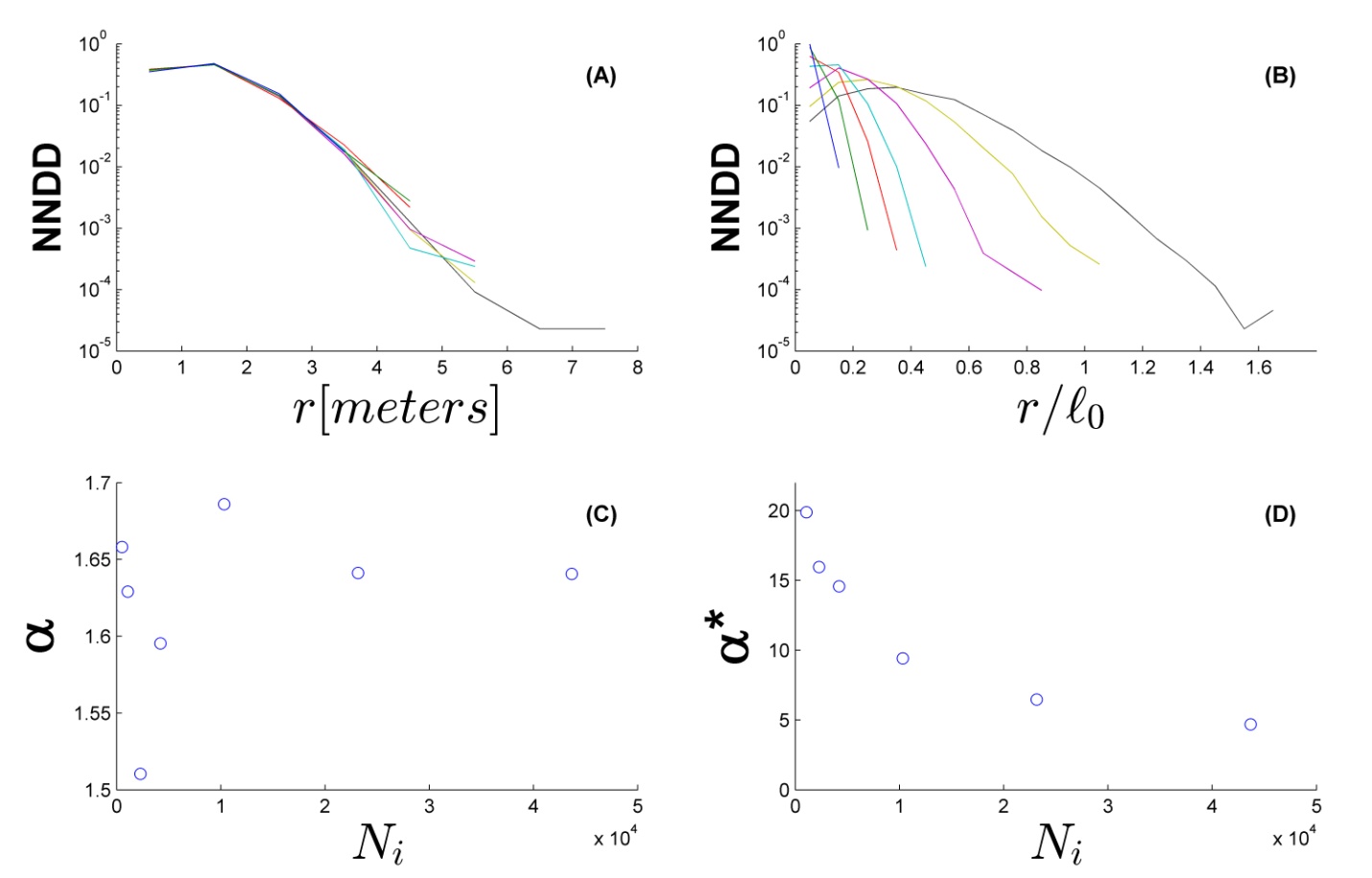


Figure 2.4: Same as fig. 2.1, here for the Random Cantor Set (P=0.75). The algorithm that generates a fractal forest, as described in section 1, yields almost no "singletons". i.e. the nearest neighbor of almost any tree is at a fixed distance. To get results that allow for a reasonable comparison with the real data we have added a weak Poissonian noise to the random Cantor set. This procedure was used only for the NNDD. The corresponding results below, for correlations and cluster statistics, were obtained for a fractal forest without any noise.

# Correlations

The correlation function for a Poisson forest (Figure 3.1) is distance independent, as opposed to the power-law decay observed in empirical data. The MLGK (3.2) shows, like in the NNDD case, a crossover to Poisson at large distances, but have a well-defined order at r=10m and r=0.5l_0_. The same holds for the Cauchy forest (3.3) and for the fractal forest (3.4).


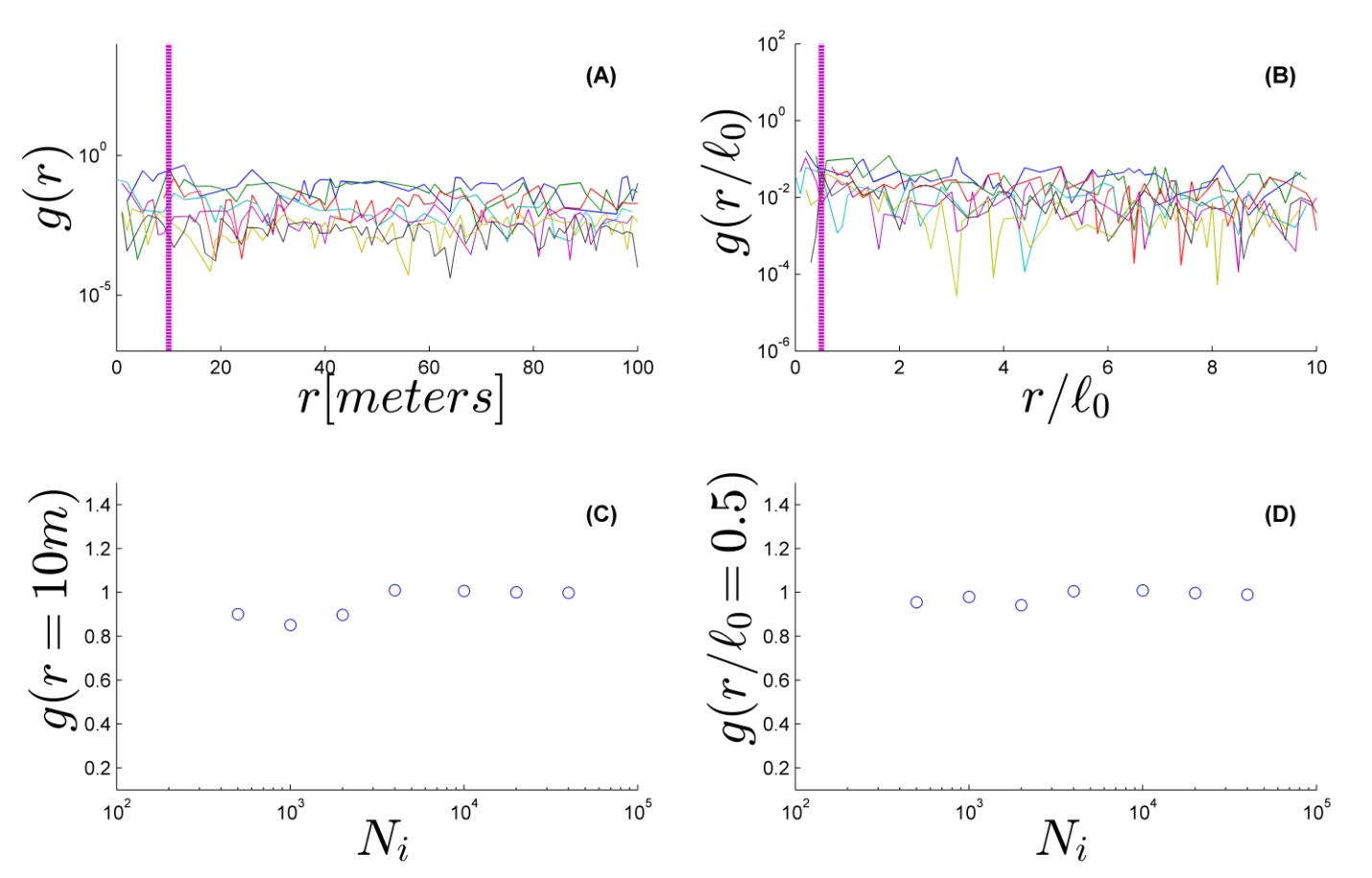


Figure 3.1: The correlation function for a Poisson forest. (A) as a function of real distance r. (B) as a function of normalized distance r/l_0_. (C) The height of the correlation function at $r=10m$is plotted against the abundance. (D) The height of $g(r/l_{0}=0.5)$ vs. $N_{i}$.


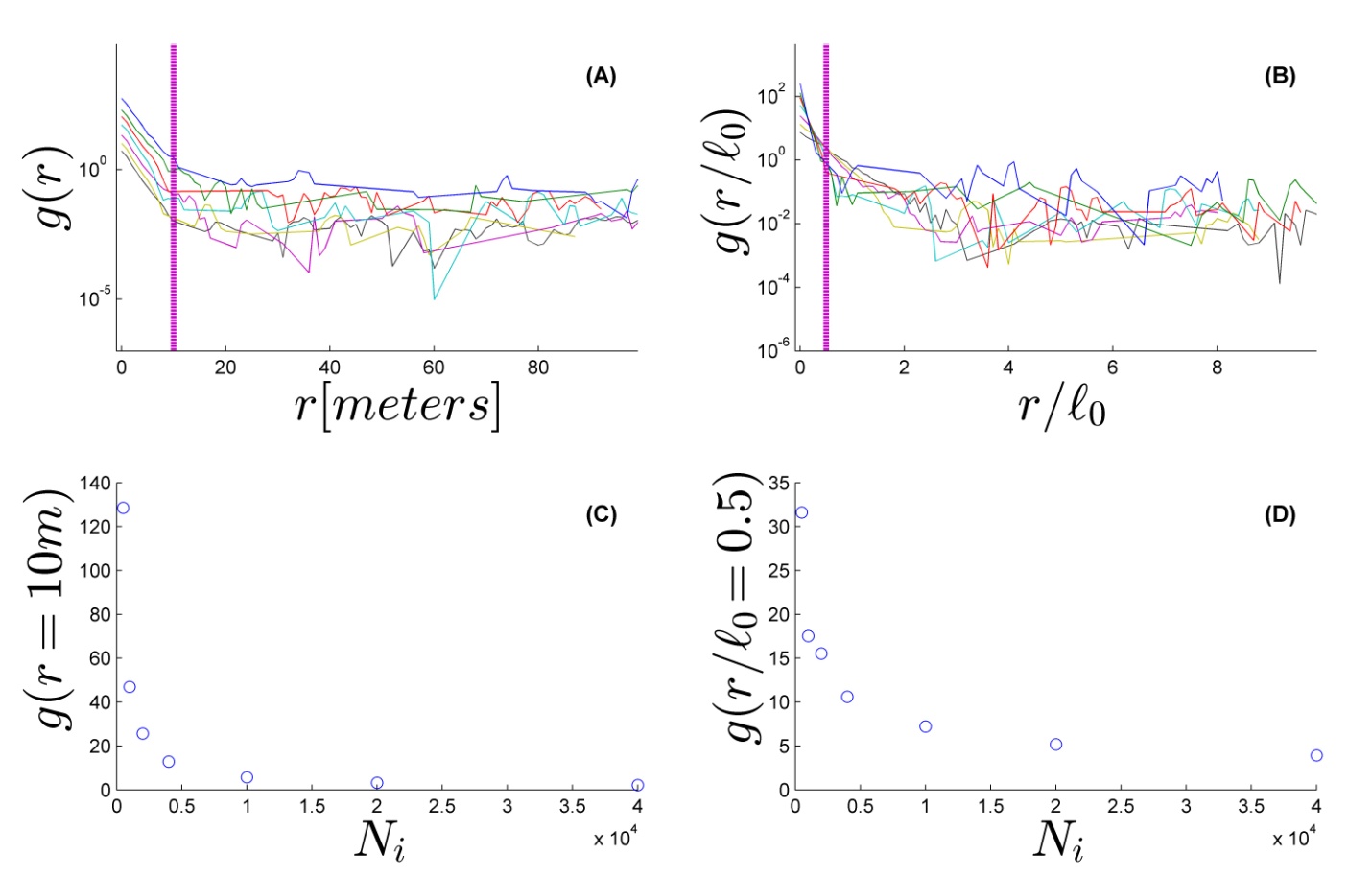


Figure 3.2: Same as fig. 3.1 but for MLGK (µ=0.1).


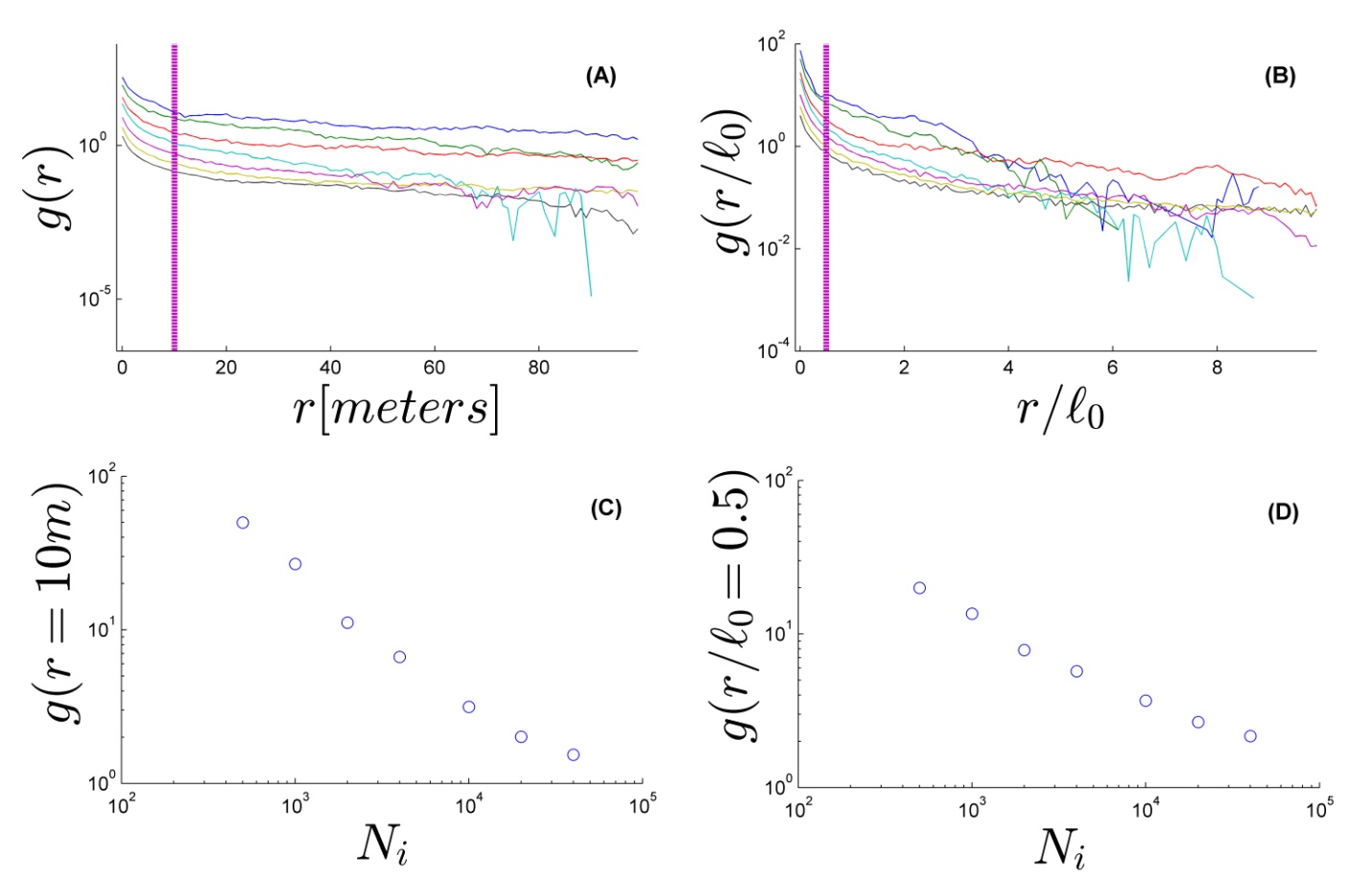


Figure 3.3: Same as fig. 3.1, here for Cauchy kernel (ɣ=20)


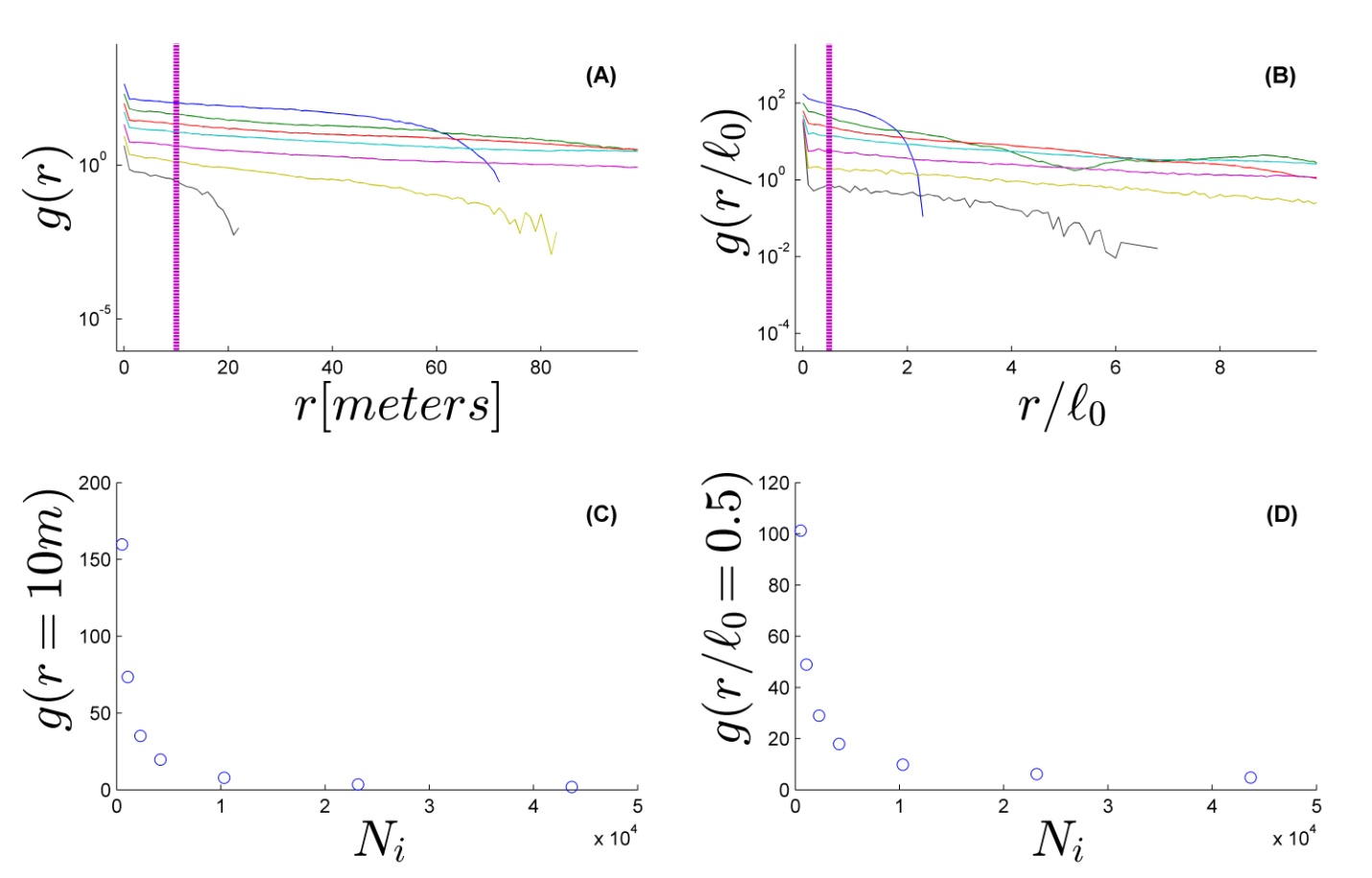


Figure 3.4: Same as fig. 3.1 now for Random Cantor Set (P=0.75).

# Patch statistics at different scales:

A graph showing F(l) vs. l has to be abundance independent when the system admit only one length scale. This property holds for the Poisson forest as depicted in Fig. 4.1. Still, the patch statistics in renormalized coordinates for a Poisson forest differs strongly from the empirical results, since the Poisson forest has no real clusters. Figure 4.5 shows together panel (B) of 4.1 and the BCI results from panel (B) of Fig. 3 of the main text, and one can see that there is no overlap between the two clusters/scale graphs.

The fractal forest shows some degree of a collapse in the normalized scales (Fig. 4.2), but the functional dependence on l is convex, unlike the concave line that characterizes the real data. In the MLGK (Fig. 4.2) and the Cauchy process (Figure 4.4) there is no collapse at all.


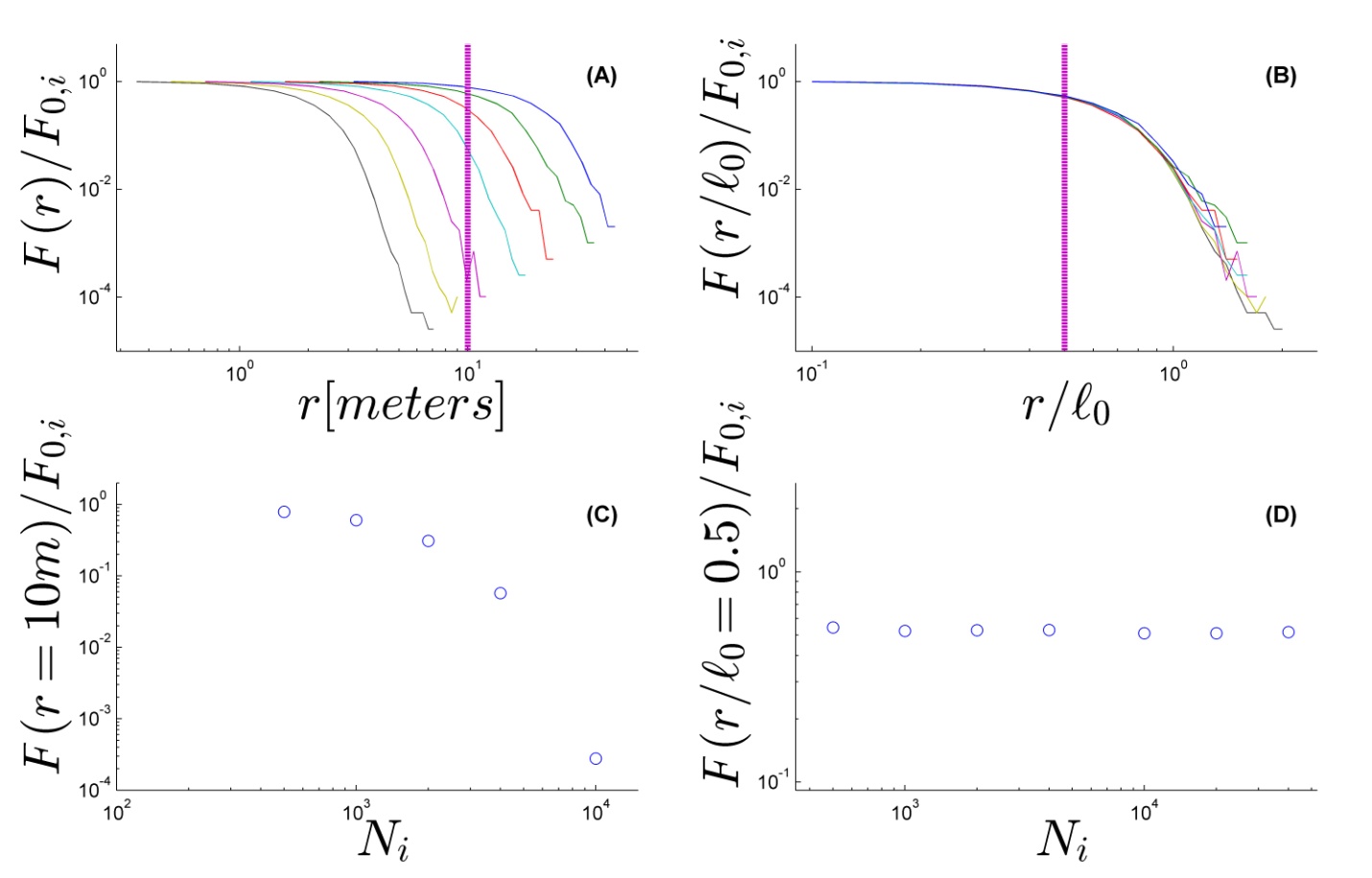


Figure 4.1: Cluster statistics for Poisson distribution. (A) as a function of real distances, r. (B) as a function of normalized distances r/l_0_. (C) $F(r=10m)/F_{0,i}$ vs. $N_{i}$ (a cut along the purple dashed line in panel (A)), (D) $F(r/l_{0}=0.5)/F_{0,i}$ vs. $N_{i}$. As expected, in (D) the correlation with the abundance disappears.


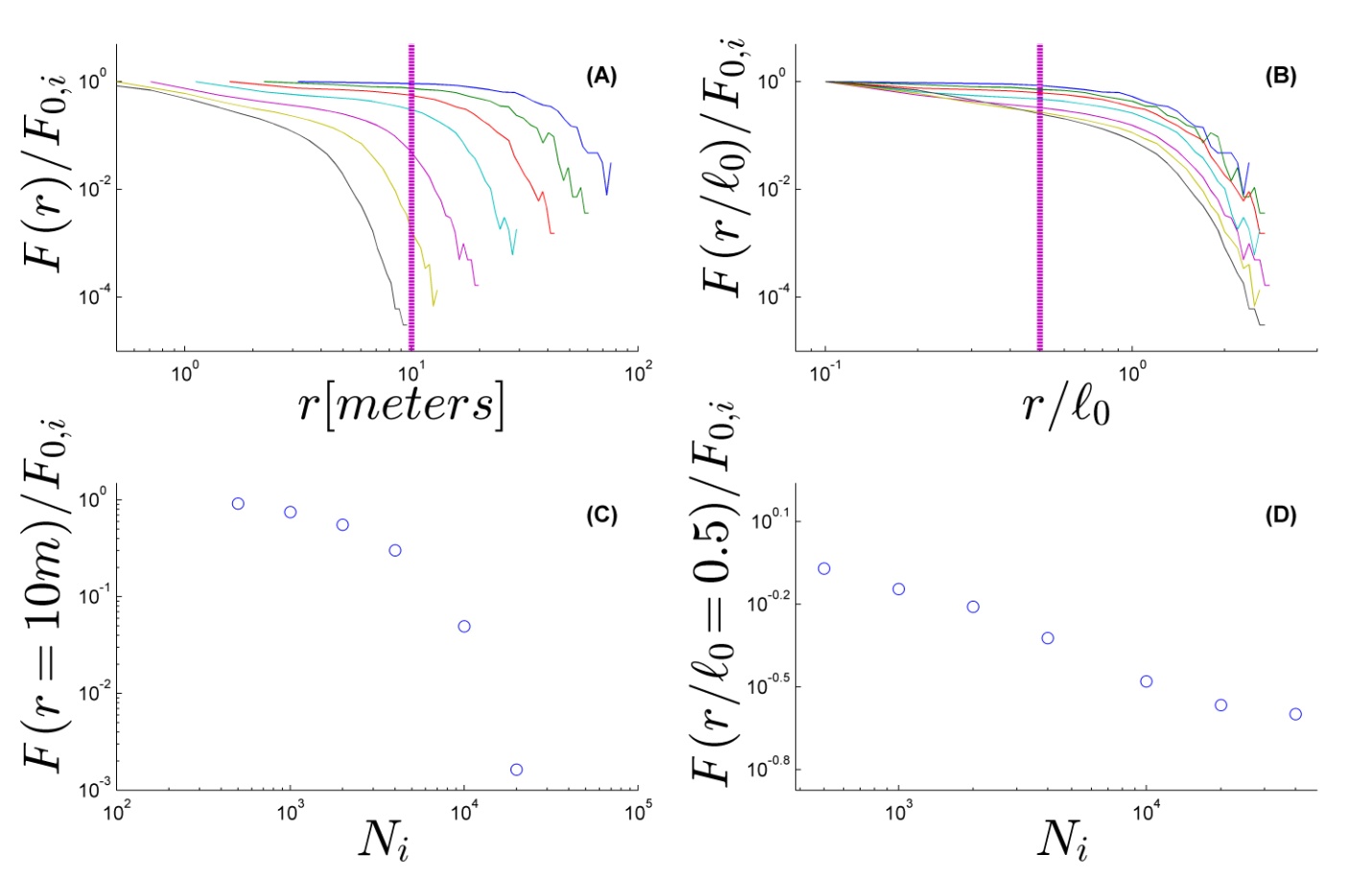


Figure 4.2: Same as fig. 4.1 for MLGK (µ=0.1).


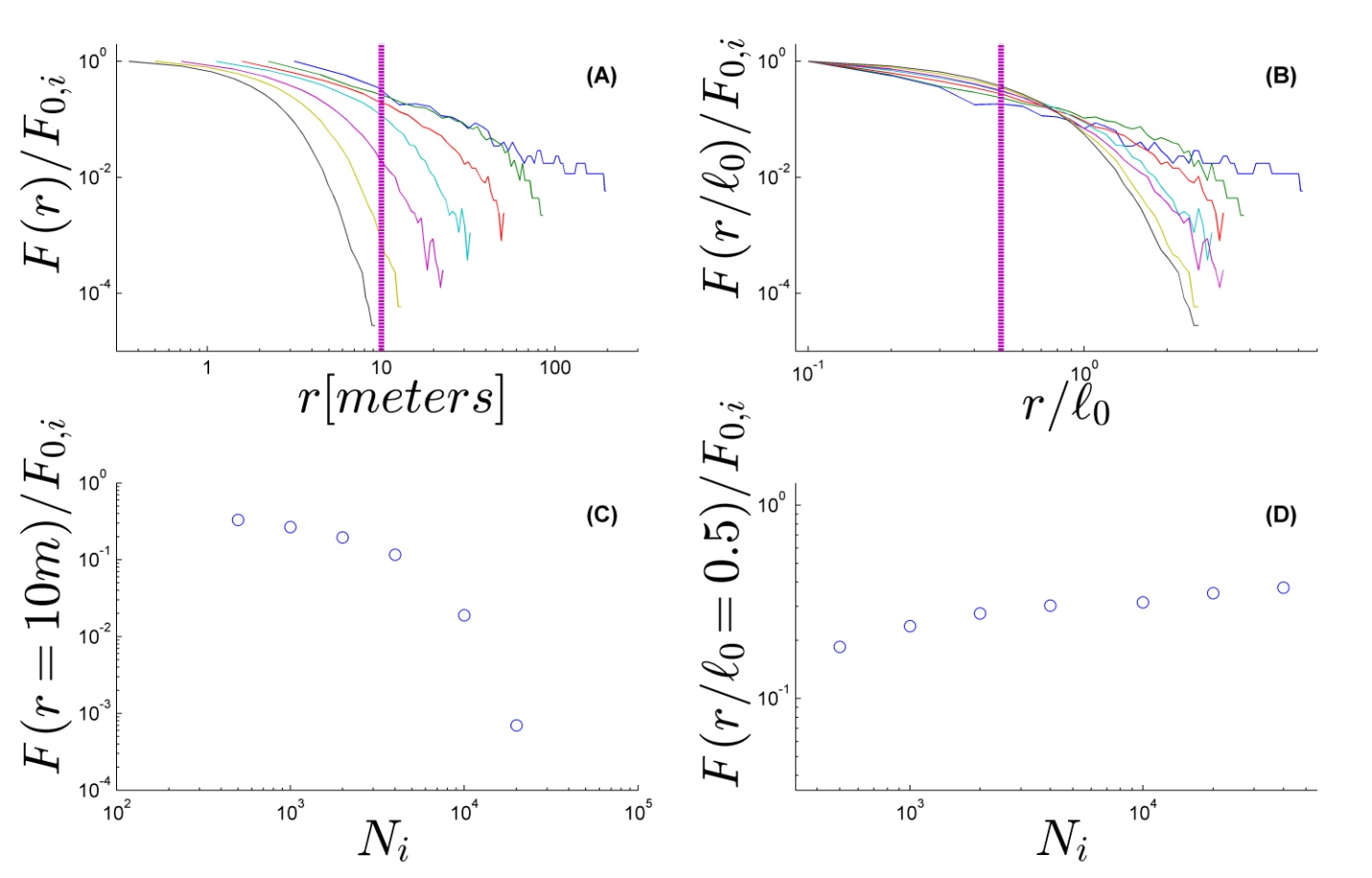


Figure 4.3: Same as fig. 4.1 for Cauchy kernel (ɣ=20)


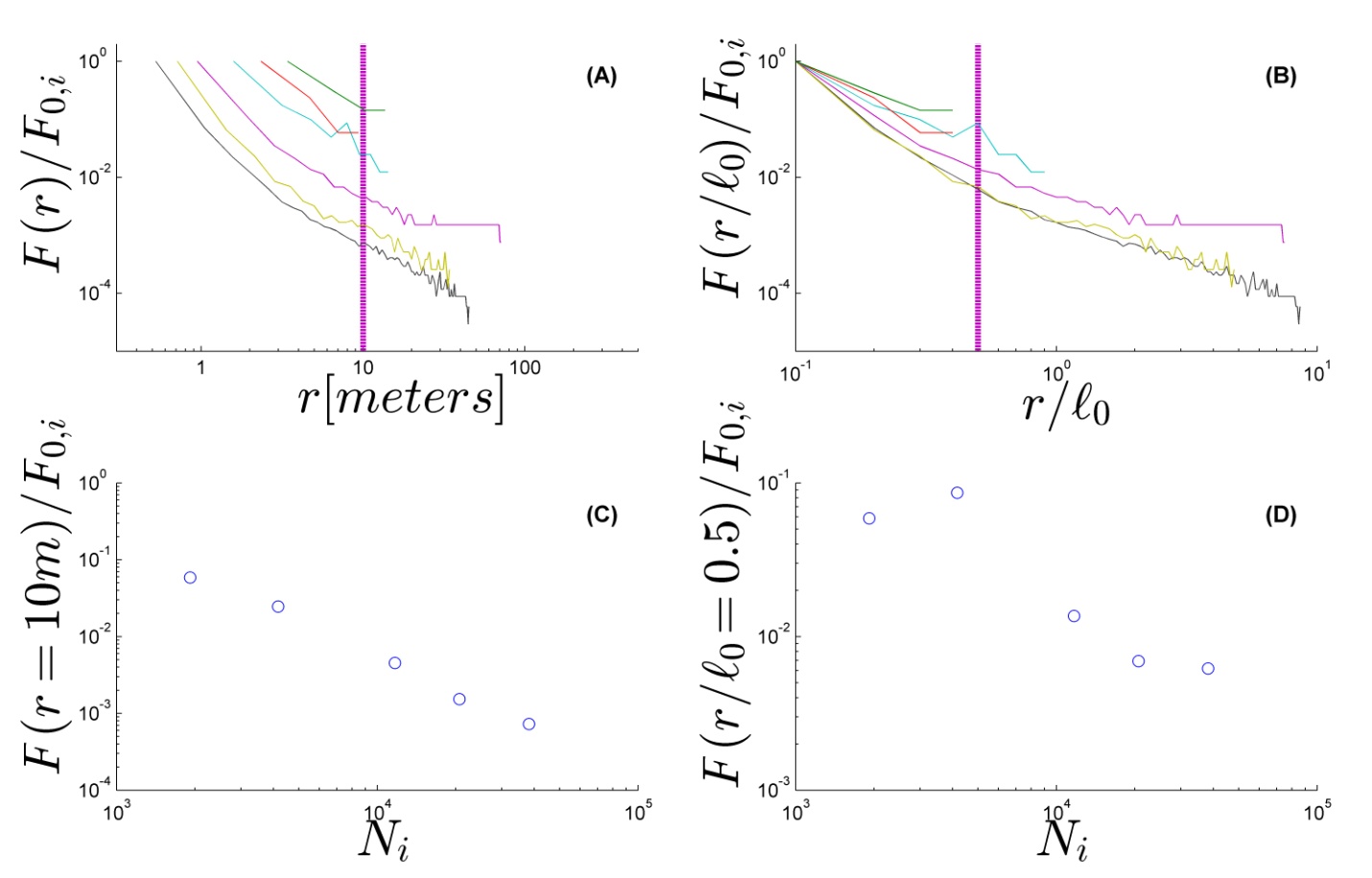


Figure 4.4: Same as fig. 4.1 for Random Cantor Set (P=0.75).

Figure 4.5: $F(r/l_{0})/F_{0,i}$ for Poisson distribution (black lines) and for BCI species (green lines).

# Exceptional species

These figures show the spatial patterns of three exceptional species in the BCI, see main text. Every red circle corresponds to >1cm individual of the focal species.


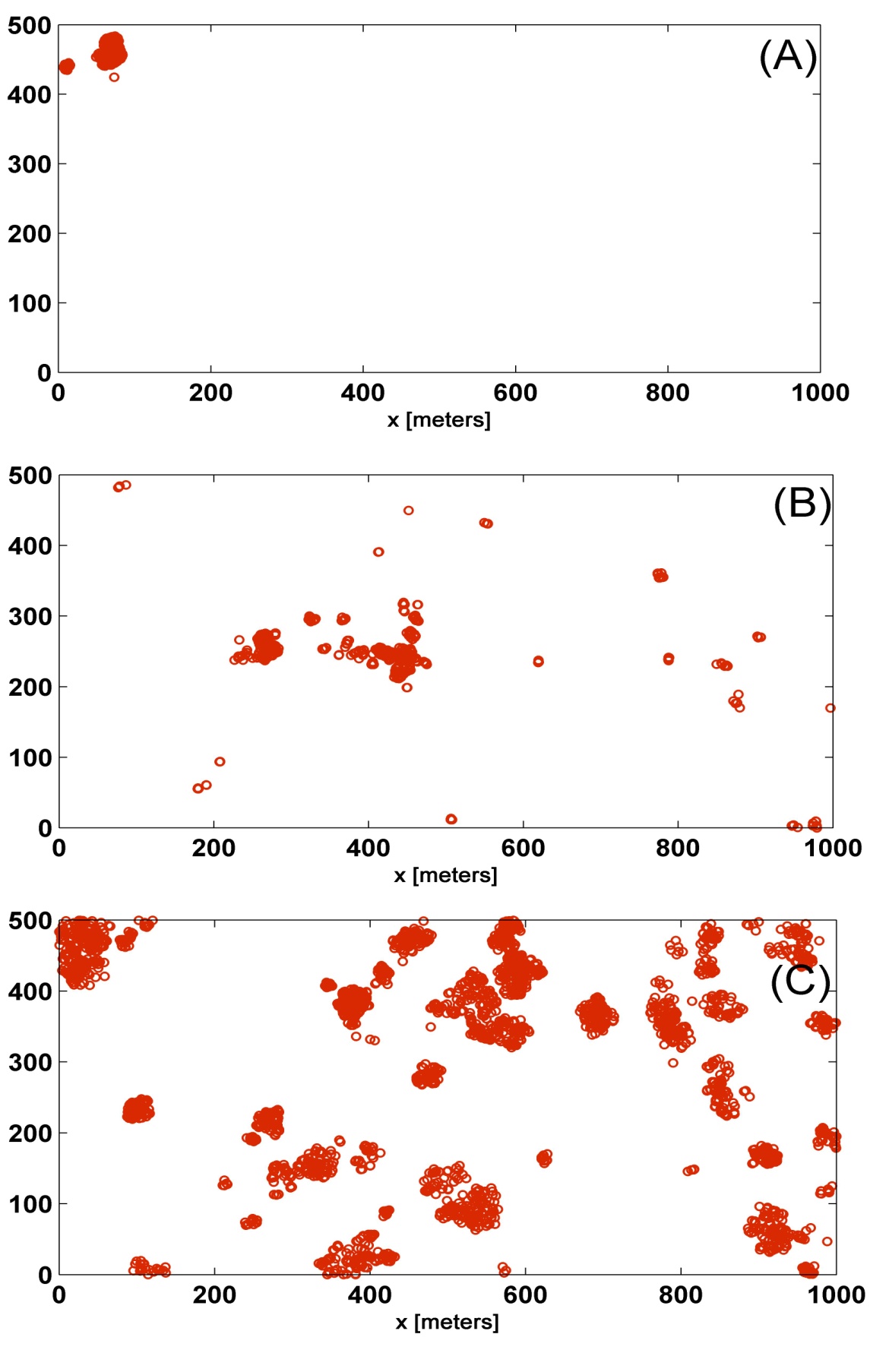


Figure 14: Exceptional species (A) Anaxagorea panamensis (B) Bactris major (C) Rinorea sylvatica.

# References

Seri, E., Y. E. Maruvka, and N. M. Shnerb. 2012. Neutral Dynamics and Cluster Statistics in a Tropical Forest. American Naturalist **180**:E161-E173.

Volkov, I., J. R. Banavar, S. P. Hubbell, and A. Maritan. 2003. Neutral theory and relative species abundance in ecology. Nature **424**:1035-1037.
